# Challenging response latencies in faking detection: The case of few items and no warnings

**DOI:** 10.3758/s13428-021-01636-z

**Published:** 2021-06-25

**Authors:** Jessica Röhner, Ronald R. Holden

**Affiliations:** 1 Department of Psychology, Otto-Friedrich-Universität Bamberg, D-96045 Bamberg, Germany; 2 Department of Psychology, Queen’s University, Kingston, Canada

**Keywords:** Congruence model of faking, Faking detection, Response times, Self-report measures

## Abstract

Abstract

Faking detection is an ongoing challenge in psychological assessment. A notable approach for detecting fakers involves the inspection of response latencies and is based on the congruence model of faking. According to this model, respondents who fake good will provide favorable responses (i.e., congruent answers) faster than they provide unfavorable (i.e., incongruent) responses. Although the model has been validated in various experimental faking studies, to date, research supporting the congruence model has focused on scales with large numbers of items. Furthermore, in this previous research, fakers have usually been warned that faking could be detected. In view of the trend to use increasingly shorter scales in assessment, it becomes important to investigate whether the congruence model also applies to self-report measures with small numbers of items. In addition, it is unclear whether warning participants about faking detection is necessary for a successful application of the congruence model. To address these issues, we reanalyzed data sets of two studies that investigated faking good and faking bad on extraversion (*n* = 255) and need for cognition (*n* = 146) scales. Reanalyses demonstrated that having only a few items per scale and not warning participants represent a challenge for the congruence model. The congruence model of faking was only partly confirmed under such conditions. Although faking good on extraversion was associated with the expected longer latencies for incongruent answers, all other conditions remained nonsignificant. Thus, properties of the measurement and properties of the procedure affect the successful application of the congruence model.

Faking can be defined as “… a response set aimed at providing a portrayal of the self that helps a person to achieve personal goals. Faking occurs when this response set is activated by situational demands and person characteristics to produce systematic differences in test scores that are not caused by the attribute of interest” (Ziegler et al., 2012, p. 8). There are additional concepts that specify the goal and the context of faking (e.g., Röhner & Schütz, 2019). For example, in personality assessment, a distinction is made between faking good (i.e., trying to make a good impression) and faking bad (i.e., trying to make a bad impression).

In recent years, researchers have paid increasing attention to the problem of faking in psychological measurement. Much of the research has highlighted that faking on psychological measures can be a serious issue, because people do fake (e.g., Birkeland et al., 2006; Viswesvaran & Ones, 1999), and faking has an influence on scale means (e.g., Rosse et al., 1998; Stark et al., 2001; Viswesvaran & Ones, 1999), rank orders (e.g., Christiansen et al., 1994; Rosse et al., 1998), and the validity of test scores (e.g., Bäckström et al., 2009). Furthermore, measures that were considered to be supposedly immune to faking have turned out to be fakeable (e.g., Röhner et al., 2011; Röhner & Ewers, 2016; Röhner & Lai, 2020). Given the potential detrimental consequences of faking, it is not surprising that researchers have spent considerable time investigating methods for detecting such behavior.

## Response latencies as a method of faking detection

A traditional method that has been investigated for the detection of faking is through the analysis of response latencies. The rationale behind this approach is that fakers, in contrast to non-fakers, differ in the amount of time they take to respond to psychological measures. A popular, albeit simplified, assumption can be summarized as: “Lying takes time” (e.g., Suchotzki et al., 2017). Being considered as an effortful process that requires extra cognitive processing and editing, it is quite intuitive that faking, in contrast to non-faking, results in longer response times. However, research has produced contradictory findings. On one hand, findings have indicated that faking is associated with longer response times (e.g., McDaniel & Timm, 1990), while on the other hand, research has also demonstrated that faking is related to shorter response times (e.g., George, 1990).

Derived from schema theory, Holden et al. (1992) have articulated a complex model of faking indicating that whether fakers respond faster or slower depends on the congruence between the generated response and the faking schema (see also Holden, 1995). According to this model, people who fake good will provide favorable responses (i.e., congruent answers) faster than they provide unfavorable (i.e., incongruent) responses. Complementarily, people who fake bad will provide unfavorable (i.e., congruent) responses faster than they will provide favorable (i.e., incongruent) responses. Thus, schema-incongruent responding is slower than schema-congruent responding. One might question why fakers provide answers that are incongruent with their faking schema. Holden and Lambert (2015) argue that faking can be a nuanced and sophisticated process rather than simple, naïve answering, especially if fakers seek to avoid presenting an extremely obvious and detectable faking pattern.

### Challenging the congruence model of faking

In general, can the latencies that individuals take to respond to psychological measures indicate whether or not they faked? According to recent meta-analyses (Maricuțoiu & Sârbescu, 2016; Suchotzki et al., 2017), response times can be informative, but how informative depends on their measurement properties. The results of the meta-analysis by Maricuțoiu and Sârbescu (2016) clearly revealed that there are differences in response times between faking good and honest responding and between faking bad and honest responding. However, they point to properties of the measurement that impact response latencies by demonstrating moderator effects of item type (i.e., larger effects computed on response latencies of positively keyed items as compared to response latencies of negatively keyed items). In the meta-analysis by Suchotzki et al. (2017), results also clearly revealed the expected and large differences in response times between faking and honest responding. However, they also point to properties of the measurement that impact response latencies. For example, the type of faking instruction played a role (i.e., whether or not participants received instructions to avoid being detected as fakers impacted their response times). Further, the to-be-faked test played a role (i.e., autobiographical Implicit Association Test produced smaller effects than the Concealed Information Test, the Sheffield Lie Test, and the Differentiation of Deception paradigm). In addition, the authors point to the fact that, like other deception measures, response time-based deception measures may be susceptible to countermeasures. Thus, the authors conclude that response time-based measures of deception have potential information, but still more research is needed. Summing up the results of both meta-analyses, the properties of the measurement (e.g., faking direction, number of items, construct to be faked, warnings) are relevant factors that need to be taken into consideration.

Empirical support for the congruence model has been found in a variety of experimental studies ranging from induced faking with university students (Brunetti et al., 1998; Esser & Schneider, 1998; Holden et al., 1992), to incarcerated offenders (Holden & Kroner, 1992), and unemployed persons who are actively seeking employment (Holden, 1998). However, in virtually all these investigations, the scales examined included 60 or more items. Considering the ongoing trend in big data analyses to use increasingly shorter scales in research, it becomes important to investigate whether the congruence model can be applied to self-report measures with smaller numbers of items. For economic reasons and also for time efficiency, research is steadily migrating toward using as few items as possible (e.g., Rammstedt & Beierlein, 2014). Whether the congruence model successfully applies in such instances, however, has not yet been evaluated. Furthermore, in addition to using a substantial number of items, most previous research involving the congruence model has warned participants that faking can be detected and that they should avoid activating any detection systems. Research has already indicated that warning participants that faking can be identified impacts participants’ responding (Dwight & Donovan, 2003). On one hand, previous research (e.g., Ben-Shakhar & Elaad, 2003; Meijer et al., 2014) has revealed larger effects on response latencies when participants received an extra incentive to avoid detection—following from this, the effects of warnings on response latencies could be a result of the motivation to avoid detection, something that is absent under non-warned conditions. On the other hand, warnings also could lead to more and better attempts to counteract faking detection (e.g., Walczyk et al., 2014)—following from this, the effects of warnings on response latencies may be even greater in non-warned conditions. A recent meta-analysis by Suchotzki et al. (2017) points to a small but nonsignificant effect of warnings on response latencies.

However, whether the inclusion of a warning is a necessary aspect of the congruence model is currently unclear. This is not trivial, because in applied settings it may not be best practice to inform test-takers that their faking can be detected. This could be both because valid faking detection methods are still missing for most psychological tests, and because respondents may not believe in the quality of detection methods. Further, warnings could have negative side effects (Robson et al., 2008) that may keep test users from utilizing them. For example, warnings may decrease the perceived test quality or increase test anxiety in test-takers (e.g., Burns et al. 2015; see also Röhner & Schütz, 2019). In this regard, recent research by Li et al. (2021) has demonstrated that warnings can induce different emotions in participants (i.e., guilt, fear, anger). These emotions are related to different outcomes. While guilt was related to a desired outcome of warnings (i.e., guilt boosted the personality scores of fakers to accuracy), fear and anger were related to negative side effects of warnings (i.e., fear led to overcorrection of non-fakers, anger reduced the perceived test fairness by fakers and non-fakers). Thus, there may be at least three reasons to avoid the use of warnings in applied settings.

## Present study

Particularly because of the use of short scales without faking detection warnings in applied settings, this research seeks to challenge the congruence model of faking (Holden et al., 1992) by using scales that include only a few items and by not warning participants about faking detection measures. Toward this goal, we reanalyze two data sets involving faked and non-faked self-description measures of extraversion and need for cognition. Extraversion was chosen because of its use in previous faking research and because, for this construct, both faking directions (i.e., good and bad) are plausible (e.g., McDaniel et al., 2009; Röhner et al., 2013; Röhner & Thoss, 2018; Steffens, 2004). Further, consistent with the congruence model, previous research has demonstrated that faking involving an extraversion scale can be detected using response latencies, at least when 60 items are used and when participants are warned about faking detection indicators (Holden & Lambert, 2015). In addition, we chose need for cognition because this construct has not been previously examined in such research. However, given the research that has demonstrated small but positive associations between need for cognition and socially desirable responding (e.g., Cacioppo et al., 1996; de Holanda Coelho et al., 2020), it is a construct that may encourage respondents to answer in a manner that makes them seem more interested in thinking in order to impress others. Overall, therefore, our study’s research focus is summarized as an investigation of whether the congruence model of faking is applicable to scales having only a small number of items and where test-takers are not warned regarding the presence of faking indicators.

## Method

### Data sets

To examine whether response latencies differ between fakers and non-fakers under the abovementioned challenging conditions, we reanalyzed data sets that were previously collected under the supervision of the lead author in an investigation of faking on scales measuring extraversion (Klink, 2018) and both extraversion and need for cognition (Hütten, 2018; Möller, 2017). We chose these data sets for several reasons: First, data sets were from studies that included both faking good and faking bad instructions. Because our interest was on the impact of faking good and of faking bad, it was necessary that both faking directions were contained in the same data set. Second, the scales used in these studies met our precondition of including comparably few items (i.e., extraversion scale: 12 items; need for cognition scale: 16 items). Third, in these studies, participants were not warned that faking could be detected, something that was a precondition for our reanalyses. Finally, because 255 (i.e., extraversion) and 146 (i.e., need for cognition) participants were included in these studies, power analyses using G*Power 3.1.7 (Faul et al., 2007) revealed a power > .99 for ANOVAs concerning the manipulation check analyses to detect a moderate effect size at an alpha level of .05 and revealed a power of > .99 (i.e., extraversion; *N* = 130) and >. 91 (i.e., need for cognition; *N* = 86) for ANOVAs concerning the response latency analyses to detect the expected large effect size at an alpha level of .05.^1^

Participants took part in the studies in exchange for personal feedback and/or partial university course credit. In both studies, individuals completed the extraversion scale (Study 1 and 2; Borkenau & Ostendorf, 2008) and/or the need for cognition scale (Study 2 only; Bless et al., 1994) twice. On the first occasion (i.e., baseline), participants completed the measures under standard instructions. On the second occasion, individuals were randomly assigned to one of three conditions (i.e., control, faking good, or faking bad). Participants in the control condition again responded under standard instruction. Fakers were asked to fake either high scores or low scores on the measures according to a personnel selection scenario. To assess the faking behavior of participants as would normally occur within a personnel context, fakers were not provided with any strategies on how to fake (i.e., *naïve faking;* see, e.g., Röhner et al., 2013, for further information), nor were they informed of the presence of any faking detection measures. In the instructions for faking good, participants were asked to imagine they had been unemployed for one year and had now received a very attractive job offer. They were asked to fake high on extraversion (and/or need for cognition) in order to maximize the chances of being offered the job. The instructions for faking bad included the description of a very unattractive job offer. To avoid being offered the job, participants were asked to fake low extraversion (and/or need for cognition).

For our analyses, we combined the data on the extraversion scale from both studies. Thus, the final sample for the extraversion scale consisted of 255 participants (93 faking bad, 86 control, 76 faking good; 186 women, 68 men, 1 no response; 251 students) with an average age of 22.34 years (*SD* = 4.59). The overall gender ratio was 72.9% women, 26.7% men, and 0.4% no response, which was very comparable to the gender ratios in subgroups (faking low: 75.3% women, 24.7% men; control group: 76.7% women, 23.3% men; faking high: 65.8% women, 32.9% men, and 1.3% no response). The final sample for the need for cognition scale consisted of 146 participants (51 faking bad, 47 control, 48 faking good; 110 women, 36 men; 145 students) with an average age of 21.89 years (*SD* = 4.34). The overall gender ratio was 75.3% women, 24.0% men, and 0.7% no response, which was very comparable to the gender ratios in subgroups (faking low: 74.5% women, 23.5% men, and 2.0% no response; control group: 80.9% women, 19.1% men; faking high: 70.8% women, 29.2% men). These data were used for the manipulation check analyses.

Concerning the response latency analyses, these data had to be checked for whether the inclusion criterion of having both high and low responses was met. After the exclusion of participants who did not fulfill this criterion, the data on the extraversion scale consisted of 130 participants (41 faking bad, 69 control, 20 faking good). The overall gender ratio was 73.8% women, 26.2% men, which, with the exception of faking high, was comparable to the gender ratios in subgroups (faking low: 73.2% women, 26.8% men; control group: 81.2% women, 18.8% men; faking high: 50.0% women, 50.0% men). The data on the need for cognition scale consisted of 86 participants (26 faking bad, 40 control, 20 faking good). The overall gender ratio was 76.7% women, 22.1% men, and 1.2% no response, which was comparable to the gender ratios in subgroups (faking low: 76.7% women, 23.1% men, and 3.8% no response; control group: 85.0% women, 15.0% men; faking high: 65.0% women, 35.0% men).

## Measures

### Extraversion scale

Participants completed the extraversion scale of the German adaptation of the NEO-Five Factor Inventory (Borkenau & Ostendorf, 2008; English version: Costa & McCrae, 1992). It consists of 12 items that are answered on 5-point ratings ranging from 0 (“strongly disagree”) to 4 (“strongly agree”). Scale characteristics in the present study were: *M =* 27.74, *SD =* 43.08; coefficient α *=* .83 at baseline assessment and *M =* 25.76, *SD =* 204.83; coefficient α *=* .96 under faking/retest.

### Need for cognition scale

Participants completed the German adaptation of the 16-item need for cognition scale (Bless et al., 1994; English version: Cacioppo & Petty, 1982). It consists of 16 items that are answered on 7-point ratings ranging from −3 (“strongly disagree”) to +3 (“strongly agree”). Scale characteristics in the present study were: *M =* 15.73, *SD =* 11.31; coefficient α *=* .85 at baseline assessment and *M =* 5.64, *SD =* 986.19; coefficient α *=* .98 under faking/retest.

## Analytical strategy

Data, syntaxes, and the outputs of our analyses are available at the OSF (https://osf.io/bh98z/).

### Manipulation check

Prior to analyzing the response latencies of fakers and comparing them to non-fakers, we verified whether the participants in the faking conditions had followed the faking instructions. To do so, we used repeated-measures ANOVAs on the individual participants’ mean scale scores of the extraversion and need for cognition scales (see e.g., Röhner et al., 2011).

### Preparation and analysis of response latencies

Separately for each scale, and based on previous procedures (Holden, 1998; Holden et al., 1992) as explicitly outlined in Paulhus and Holden (2010), raw item response times for the second administration of items were adjusted to control for the effects of statistical outliers and were twice standardized, once to adjust for confounding respondent factors (e.g., reading speed, sex) and a second time to adjust for confounding item variables (e.g., length, vocabulary level). This involved the following: First, to mitigate the influence of statistical outliers, response latencies of less than 0.5 s or greater than 40 s were set to 0.5 or 40 s, respectively. Second, response latencies were standardized across items, within each participant, to control for irrelevant differences between individual respondents. Third, response times were standardized across participants within each item to adjust for differences between items. Of note, the means and standard deviations used for this second standardization were from the corresponding items for all participants during the baseline administration (i.e., prior to the experimental assignment to different instructional conditions). Fourth, response times were readjusted for outliers, such that latencies of less than −3.00 or greater than 3.00 were set to −3.00 or 3.00, respectively. Overall, this preparation procedure yields response latencies that are calculated both relative to the respondent and relative to the scale item, and are unconfounded by main effect influences of specific persons, specific items, and statistical outliers. Following this preparation, these adjusted latencies were aggregated for each respondent to produce means for high scores on items (scores of 3 or 4 on extraversion items; scores of 1, 2, or 3 on need for cognition items) and low scores on items (scores of 0 or 1 on extraversion items; scores of −3, −2, or −1 on need for cognition items.). For each participant, these two mean adjusted response latency scores were the units of analysis for each scale. Item latencies for “neutral” responses (i.e., scored 2 on extraversion items; scored 0 on need for cognition items) were excluded from the analyses. ANOVAs then were used to compare mean adjusted response latencies for the control, faking good, and faking bad groups.

## Results

### Manipulation check

As expected, ANOVAs revealed that participants were motivated and well able to fake high and low scores on the extraversion scale and on the need for cognition scale. A 2 (measurement occasion) × 3 (experimental group) ANOVA with repeated measures on the first factor and the scores on the extraversion scale as the dependent variable indicated that participants in the faking groups faked according to their faking instructions on the extraversion scale (see Table 1). The significant main effect of group, *F*(2, 252) = 165.96, *p* < .001, ω^2^ = .56, was qualified by the expected large and significant interaction effect, *F*(2, 252) = 428.88, *p* < .001, ω^2^ = .77. The main effect of measurement occasion, *F*(1, 252) = 3.77, *p* = .053, ω^2^ = .01, was nonsignificant. A similar 2 (measurement occasion) × 3 (experimental group) ANOVA for scores on the need for cognition scale also confirmed that participants faked according to their instructions for the need for cognition scale (see Table 1). The significant main effects of measurement occasion, *F*(1, 143) = 71.92, *p* < .001, ω^2^ = .33, and group, *F*(2, 143) = 179.34, *p* < .001, ω^2^ = .71, were qualified by the expected large and significant interaction effect, *F*(2, 143) = 356.48, *p* < .001, ω^2^ = .83.

**Table 1:** Descriptive Variables and Post Hoc Comparisons Regarding the Means of the Extraversion Scale and the Need for Cognition Scale

	Measure					
	Extraversion Scale			Need For Cognition Scale		
	Experimental group			Experimental group		
	Faking bad	Control	Faking good	Faking bad	Control	Faking good
Measurement occasion	*M (SD)*	*M (SD)*	*M (SD)*	*M (SD)*	*M (SD)*	*M (SD)*
Baseline	2.37_a1_ (0.50)	2.30_a1_ (0.58)	2.25_a1_ (0.57)	0.98_a1_ (0.77)	0.98 _a1_ (0.70)	1.00 _a1_ (0.66)
Faking	0.88_b2_ (0.64)	2.33_a1_ (0.55)	3.49_c2_ (0.37)	-2.01_b2_ (0.81)	0.90 _a1_ (0.70)	2.33_c2_ (0.62)

*Note*. *N* = 255 for the extraversion scale. *N* = 146 for the need for cognition scale. Different lettered subscripts indicate significant differences between experimental groups (i.e., columns); different numbered subscripts identify significant differences between measurement occasions (i.e., rows) at *p* < .05

### Response latency analyses

According to the congruence model of faking, participants were included in the response latency analyses only if they had a latency for high responses *and* for low responses. If a participant provided no low responses or no high responses, that person was excluded from analyses. Applying this inclusion criterion led to samples of 130 (i.e., extraversion) and 86 participants (i.e., need for cognition) for the response latency analyses.

**Fig. 1:**
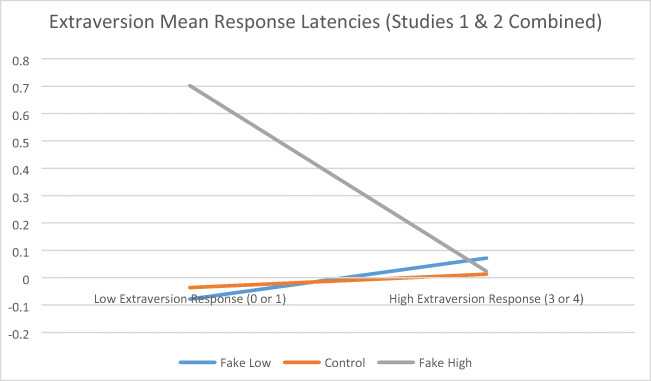
Extraversion mean response latencies (Studies 1 & 2 Combined)

**Fig. 2:**
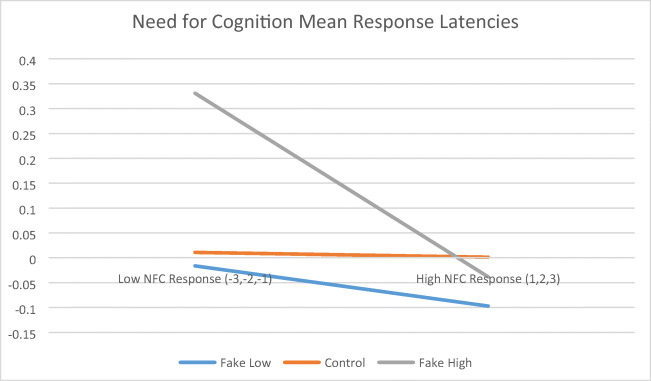
Need for cognition mean respone latencies

Mean adjusted item response latencies as a function of response and faking condition are presented for extraversion and for need for cognition scales in Table 2. For extraversion, using response type (i.e., high vs. low responses) as a within-subject factor and faking instructional group (i.e., control, faking good, faking bad) as a between-subjects factor, a significant type of response by group interaction (see Figure 1), *F*(2, 127) = 4.30, *p* < .05, partial *η*^2^ = .06, approximating a medium effect size, partially supported the Holden et al. (1992) congruence model. However, based on the Games-Howell procedure (not requiring homogeneous variances), post hoc comparisons among faking groups within each response type indicated only differences for the faking good group taking longer to provide low extraversion answers than the control group. For need for cognition, no significant response type by group interaction emerged (see Figure 2), *F*(2, 83) = 0.95, *p* > .05, partial *η*^2^ = .02.

**Table 2:** Mean (SD) Adjusted Response Latency by Response and Faking Condition

Scale		Experimental Group			
	Type of Response	Control	Faking Good	Faking Bad	*F-*ratio
Extraversion (Studies 1 and 2 Combined)	High Responses	0.00 (0.42)	-0.07 (0.20)	0.10 (1.09)	1.08
Extraversion (Studies 1 and 2 Combined)	Low Responses	-0.04 (0.75)	0.70 (1.18)	-0.04 (0.21)	12.26**
Need for cognition (Study 2)	High Responses	-0.01 (0.19)	-0.02 (0.07)	-0.10 (0.76)	0.52
Need for cognition (Study 2)	Low Responses	0.01 (0.67)	0.33 (1.28)	-0.01 (0.16)	1.98

**p < .001

*Note*. A mean latency of 0.00 for high Extraversion responses in the Control Group implies that these responses were neither faster nor slower than responses for the other experimental groups or for low Extraversion responses for any experimental group.

## Discussion

We reanalyzed two data sets in order to investigate whether the congruence model of faking could be applied successfully on scales that have comparably few items (i.e., 12 and 16) and in contexts where warnings that faking can be detected are not used. In view of the growing tendency to use shorter scales and the various approaches regarding warnings, this is a crucial question. Our results revealed that the congruence model of faking was only partly supported under these conditions (i.e., for incongruent responses to faking good on extraversion). Thus, the applicability of the model depends on the measurement procedure.

## Can response latencies indicate fakers on scales with few items in unwarned situations?

With regard to faking on the extraversion scale, our analyses revealed that, under these circumstances and in line with the congruence model of faking, the response latencies of fakers differed significantly from those of non-fakers when respondents were instructed to fake good. The differences in response latencies were limited to faking-good-incongruent answering. When faking bad was instructed, any differences in response latencies were nonsignificant. For faking on the need for cognition scale, the same pattern was found, but only at a statistically nonsignificant descriptive level. The differences between the two scales could be attributed either to the to-be-faked measure, to the number of items that were included (i.e., the need for cognition scale included four more items than the extraversion scale), or to both.

The present results align with findings from the meta-analysis by Maricotoiu and Sârbescu (2016) that revealed that differences in response latencies are more pronounced when faking good than when faking bad is instructed. The findings also support the results of recent research by Bensch et al. (2019), who explored differences between faking good and faking bad. Bensch et al. (2019) note that faking good and faking bad are related to different psychological processes. They indicate that faking good is easier than faking bad and suggest that, when faking good, it might be obvious which items are relevant to be faked and how one should respond to portray oneself in a positive way. Alternatively, when faking bad, it might also be obvious which items are relevant, but the best way to portray oneself in a negative way may be a less clear process. Their findings may assist in explaining our results. The congruence model fit the "easier" faking good direction, because respondents had to process less on how to fake, and thus differences in response times became more apparent. It could be speculated that, in children, being nice and presenting positively is something that becomes thoroughly ingrained during upbringing and is automatic in processing. The model did not fit "the more difficult" faking bad direction, because it entailed more cognitive processing of something that is not automatic, and response latencies were, thus, more comparable to what respondents do in determining an honest response that represents their personality.

Earlier research has also often warned participants that faking can be detected. In unwarned conditions, such as in the current investigation, the effects of faking on response latencies were smaller than what has been found under warned conditions (e.g., Ben-Shakhar & Elaad, 2003; Meijer et al., 2014). Thus, warnings seem to be a necessary precondition. From an applied point of view, this is relevant because warnings cannot always be included, particularly when the original development of a scale has not included warnings, and there is a desire to use testing conditions that are identical to those associated with established test normative scores. Another consideration may be the degree to which respondents believe or fail to believe in any warnings that are given. This is a direction for future research.

The issue arises as to why short scales with no warnings about faking produce a weakened effect for the congruence model. In fact, the applicability of the model depends on a respondent producing incongruent answers, responses that are discrepant from the test-taker’s goal. However, with a small number of items per scale, there was less opportunity for faking-discrepant responses to occur. Although using incongruent answers represents a nuanced faking strategy (e.g., Holden & Lambert, 2015) designed to avoid the detection of obvious faking, not including warnings might decrease the use of such nuanced faking. Thus, in addition to less opportunity to use incongruent answers, it might also be possible that with no warnings present, more elaborate strategies of faking are not invoked because respondents are less concerned about being caught faking. Of note are the relatively few degrees of freedom associated with the *F*-ratios of the mean response latencies reported for the interaction of type of response with group congruence. Using the extraversion scale as an example, although there were 255 participants, 125 of them did not provide both low *and* high responses for the 12 items of that scale and, thus, because mean adjusted latencies could not be calculated, could not be included in the analysis of response times. With more items and a warning about faking detection, a larger number of faking-discrepant responses would have resulted in stronger support for the congruence model that contrasts congruent and incongruent responding.

## Limitations

Our study has potential limitations regarding the applicability of the congruence model. First, we examined only two constructs (i.e., extraversion and need for cognition). Future research should investigate the applicability of the congruence model of faking using other to-be-faked constructs. Second, with regard to the number of items of a measure, we used scales that involved 12 and 16 items. Previous research has focused on measures of 60 or more items. Thus, an important issue is the minimum number of items necessary to produce a meaningful result. Third, our samples primarily consisted of female (> 70%) students who were instructed to fake. Considering research that has demonstrated differences in response times between female and male respondents (e.g., Dane & Erzurumluoglu, 2003; Dykiert et al., 2012), the over-representation of women might raise a concern about the generalizability of our results. However, the double-standardization method as used in the congruence model of faking adjusts for potential confounding respondent factors (e.g., reading speed, sex) and for potential confounding item variables (e.g., length, vocabulary level). Nevertheless, whether the results are generalizable to samples from other populations (e.g., job applicants, gender-balanced) and to naturally occurring faking are avenues for future research. Fourth, in examining the congruence model of faking, our focus was on response type rather than on other potential influences such as item keying. Additional research could serve to articulate the influence of other factors that could impact on faking. Fifth, the inclusion criterion for the congruence model of faking led to the exclusion of numerous participants for the response latency analyses. Thus, the power was sufficient in each data set (above .91) to detect large effect sizes as expected from the large and significant overall average effect size found in a recent meta-analysis by Suchotzki et al. (2017). However, the power of our study was not sufficient to detect moderate or small effect sizes, as indicated by the meta-analysis by Maricuțoiu and Sârbescu (2016). We calculated our power estimation based on the results of the more recent meta-analysis by Suchotzki et al. (2017), because we believed that their effect size estimation is more reliable, for the following reasons: Suchotzki et al. (2017) included 114 studies, whereas Maricuțoiu and Sârbescu (2016) included only 16 studies; and Maricuțoiu and Sârbescu (2016) included mainly studies on faking good, which is typically less pronounced than faking bad (e.g., Röhner et al., 2011), and which in turn might have caused an underestimation of the effect size. Nevertheless, researchers interested in investigating the congruence model may thus want to ensure they have a very large number of participants and items, particularly if wanting to use the information at the individual respondent level.

## Summary and conclusion

Our results indicate that having a small number of items on a measure and not warning participants about faking detection has a deleterious effect on successfully applying the congruence model of faking. Under these conditions, the model was only partly supported. Although faking good on the extraversion scale was associated with the expected longer latencies for incongruent answers, all other conditions failed to support the model. Thus, properties of the measurement and aspects of warnings impact whether the congruence model can be applied successfully. As such, measurement conditions are an important consideration when applying response times for the detection of faking.

## Footnotes

1 According to the congruence model of faking, participants are included in the response latency analyses only if they have a latency for high responses *and* for low responses. If a participant provided no low responses or no high responses, that person is excluded from analyses. Applying this procedure of inclusion criteria led to the exclusion of numerous participants.
